# Nitric Oxide-Dependent Regulation of Cytokines Release in Type-II Diabetes Mellitus

**DOI:** 10.1155/2013/531026

**Published:** 2013-02-17

**Authors:** Maqsood M. Elahi, Bashir M. Matata

**Affiliations:** ^1^Division of Cardiothoracic Surgery, Department of Surgery, Scott & White Memorial Hospital, Texas A&M Health Science Centre, 2401 S. 31st Street, Temple, TX 76508, USA; ^2^Department of Clinical Research, The Liverpool Heart and Chest Hospital NHS Foundation Trust, Thomas Drive, Liverpool L14 3PE, UK

## Abstract

The mechanism of release of proinflammatory cytokines by blood granulocytes in diabetes is unknown. We investigated whether diabetes mellitus affects the production of cytokines by granulocytes (PMN) and mononuclear cells (PBMCs) and whether this is modulated by NO. Isolated PMN and PBMC from with or without type-II diabetes mellitus were incubated at 37°C for 6 h with S-nitroso-N-acetylpenicillamine (SNAP) at 0, 1, and 100 **μ**M with or without lipopolysaccharides (LPS) stimulation (1 **μ**g/mL). Supernatants were assayed for tumor necrosis factor-**α** (TNF-**α**) and interleukin-8 (IL-8) by sandwich ELISA. Significant increases in TNF-**α** and IL-8 were observed only in PMN from diabetic subjects with or without LPS stimulation and that exogenous NO inhibited further production of cytokines in a concentration-dependent manner. However, activity of PBMC when stimulated with LPS was greatly enhanced by diabetes, but not affected by NO production. Hence, suggesting that granulocytes activation and participation in diabetes related complications is modulated by NO bioavailability.

## 1. Introduction

Production of basal levels of superoxide (O_2_^∙−^) and nitric oxide (NO) radical by granulocytes is known to be enhanced in patients with type-II diabetes [1]. Recently it was reported in healthy subjects that NO production by granulocytes is mediated via cyclic AMP and cGMP pathways [2]. However, cyclic AMP and cGMP-elevating agents inhibited the production of NO in granulocytes isolated from human subjects with type-II diabetes, thus suggesting a possible disease-induced adapted metabolic response [2]. The consequences of this altered metabolic response on host defense and inflammation hence is unknown. Previously we demonstrated that NO-mediated pathway induce a dose-dependent release of proinflammatory cytokines in human monocytes through the modulation of nuclear factor-kappa-B-DNA binding activity [3] However, the mechanism of release of proinflammatory cytokines by blood granulocytes especially in diabetes yet remains not clear. In this study, we tend to investigate whether diabetes mellitus similarly affects the production of cytokines by granulocytes (PMN) and whether NO modulates this.

## 2. Materials and Methods

### 2.1. Reagents

The following reagents were all obtained from Sigma Biochemicals (Dorset, UK): Hanks balanced salt solution, S-nitroso-N-acetylpenicillamine (SNAP), dextran sedimentation solution, Histopaque-1077 medium, and lipopolysaccharides (LPS). 

### 2.2. Granulocytes Separation

PMNs were purified by dextran sedimentation followed by gradient density centrifugation on Histopaque-1077 at 800 g for 28 minutes. A 15 mL heparinized venous blood was obtained from healthy nonsmoking volunteers (age 52.3 ± 3.4 years) and type-II diabetic patients (age 56.4 ± 4.5 years) attending outpatient's clinic at Faculty of Medicine & Surgery, PIC. There were equal proportions of females between the healthy volunteer group and the diabetic patients (3 out of 8 per group). The local ethics committee approved the study protocol and informed consent was obtained from all participants. Patients were excluded if they were smokers, undergoing treatment for hypertension, were pregnant, and had cardiac insufficiency, inflammatory conditions, malignancy, and infection. Patients were included if they were at the age range 30–70, cutoff for fasting plasma glucose >140 mg/dL, and without prior insulin treatment. The isolation yielded up to 98% pure granulocytes populations as judged by acetic-acid-crystal violet staining and 95%–98% viability by trypan blue exclusion. A 1 × 10^6^/mL of PMNs and peripheral blood mononuclear cell fractions (PBMC) from nondiabetic healthy volunteers and type-II diabetic subjects were incubated in humidified atmosphere of 5%  CO_2_ at 37°C for 6 h in the presence of the NO donor SNAP (at concentrations of 0, 1, 100 *μ*M) with or without LPS treatment (1 *μ*g/mL). Supernatants were assayed for tumor necrosis factor-*α* (TNF-*α*) and interleukin-8 (IL-8) by sandwich ELISA (Pharmingen Inc.). 

## 3. Results

**Figure 1:**
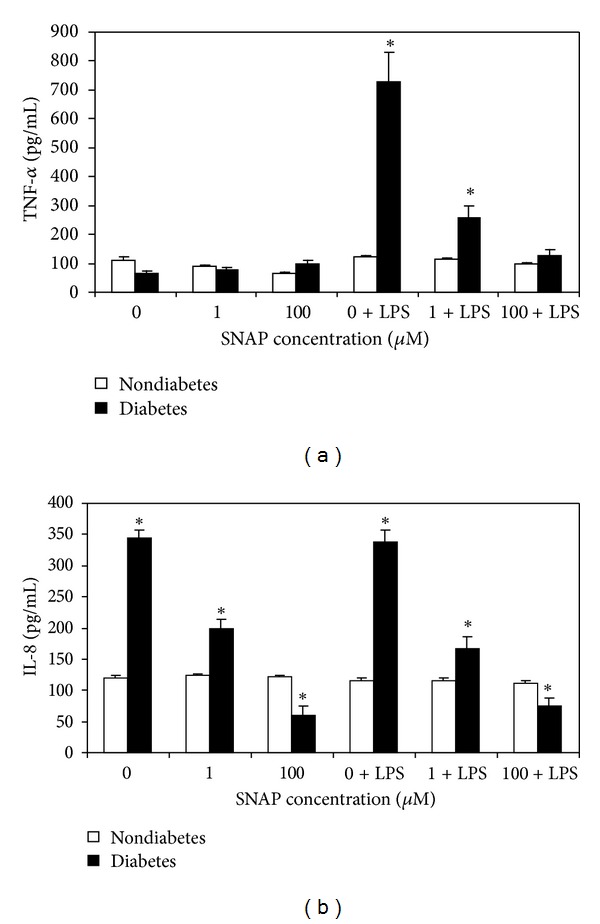
Increasing concentrations of exogenous NO significantly inhibited TNF-*α* (a) and IL-8 (b) production by granulocytes with or without LPS stimulation in patients with diabetes. Values expressed as mean ± SE, *n* = 8/group. *Represents *P* < 0.05.

**Figure 2:**
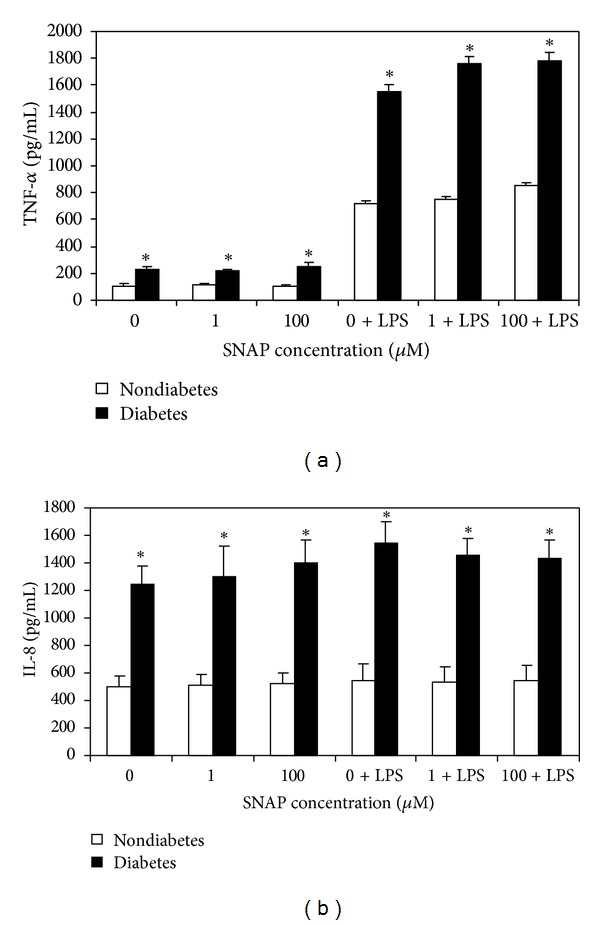
Increasing concentrations of exogenous NO had no significant effect on TNF-*α* (a) and IL-8 (b) production by peripheral blood mononuclear cells with or without LPS stimulation in patients with or without diabetes. Values expressed as mean ± SE, *n* = 8/group. *Represents *P* < 0.05.

We have demonstrated this model of isolated blood PMNs that at low concentrations of SNAP (0-1 *μ*M) production of soluble TNF-*α* was similar between diabetic and nondiabetic subjects. However, at higher SNAP concentrations (100 *μ*M) PMNs isolated from diabetic subjects produced significantly higher soluble TNF-*α* and IL-8 cytokines (Figures 1(a) and 1(b)). Interestingly, stimulation of PMNs from diabetic but not nondiabetic subjects with LPS in the presence of increasing concentrations of SNAP inhibited soluble TNF-*α* and IL-8 cytokines production in a concentration-dependent manner. In contrast, in the PBMC model, the magnitude of soluble TNF-*α* cytokines was almost 2-fold greater in the diabetic than in nondiabetic irrespective of the concentration of SNAP or LPS stimulation (Figure 2(a)). In addition, although IL-8 concentrations were 2-fold greater in diabetic PBMC, values remained the same irrespective of concentration of SNAP and LPS (Figure 2(b)). 

## 4. Discussion

Although the causes of diabetes mellitus are diverse, the end result is an alteration of the glucose metabolism. The underlying changes in leukocytes cell functions instigated by altered glucose metabolism are still a subject of much research. Previously, it has been shown that endothelial transmigration of PMNs obtained from diabetic patients with high insulin was increased compared with that of healthy subjects and that this may be accelerated by the expression of platelet endothelial cell adhesion molecule-1 [4]. However, sustained hyperglycaemia leads to endothelial dysfunction, which in turn can cause vascular lesions and a deficit of NO production coupled with increased reactive oxidant species [5, 6]. Evidence suggests that NO production by PMN from diabetic and nondiabetic subjects was modulated by cAMP and cGMP [2], yet little is known as to whether NO has any regulatory function in the inflammatory process. In this study we demonstrated that high levels of exogenous NO in a concentration-dependent manner significantly downregulated the production of TNF-*α* and IL-8 cytokines by PMN from patients with type-II diabetes following LPS stimulation which may suggest that under conditions of hyperinsulinaemia, modulation of PMN response to infection may be dependent upon NO bioavailability, a factor that may be central to the propagation of diabetes related complications.
